# Mendelian Randomization Study on the Putative Causal Effects of Omega-3 Fatty Acids on Low Back Pain

**DOI:** 10.3389/fnut.2022.819635

**Published:** 2022-02-14

**Authors:** Shan Zhou, Gaizhi Zhu, Yaqi Xu, Ran Gao, Huan Li, Gencheng Han, Wenting Su, Renxi Wang

**Affiliations:** ^1^Beijing Institute of Brain Disorders, Laboratory of Brain Disorders, Ministry of Science and Technology, Collaborative Innovation Center for Brain Disorders, Capital Medical University, Beijing, China; ^2^Department of Neuroimmune and Antibody Engineering, Beijing Institute of Basic Medical Sciences, Beijing, China

**Keywords:** low back pain, omega-3, the causal link, genome-wide association study, Mendelian randomization, single nucleotide polymorphism

## Abstract

Previous observational studies have suggested an important role of omega-3 in low back pain. In the present study, we used a two-sample Mendelian randomization (MR) study to identify the putative causal link between omega-3 and low back pain. A broadly used genome-wide association study (GWAS) (*n* = 8,866 individuals from European ancestry) was used to select plasma omega-3 genetic instrumental variables (IVs). A previously reported GWAS (4,863 cases and 74,589 controls from European ancestry) for low back pain were used to assess the effect of plasma omega-3 levels on low back pain. MR-egger_intercept, MR-PRESSO, MR_egger, and inverse variance weighted (IVW) in Cochran's *Q*-test were used to determine the pleiotropy and heterogeneity, respectively. MR-egger, weighted median, IVW, and weighted mode were used to perform MR analysis. Finally, the effect of a single nucleotide polymorphism (SNP) was used to test the SNP bias. We did not find a significant pleiotropy or heterogeneity of all six selected plasma omega-3 genetic IVs in low back pain GWAS. Expectedly, we found that as plasma omega-3 levels genetically increased, the risk of low back pain had a decreased trend using MR-egger (Beta = −0.593, *p* = 0.228; OR = 0.553) and weighted mode (Beta = −0.251, *p* = 0.281; OR = 0.778). This reduced trend was further proven by weighted median (Beta = −0.436, *p* = 0.025; OR = 0.646) and IVW (Beta = −0.366, *p* = 0.049; OR = 0.694). Our analysis suggested a putative causal link between genetically increased plasma omega-3 levels and the reduced risk of low back pain in European ancestries. Thus, the supplementation of omega-3 may be important for the prevention and treatment of low back pain.

## Introduction

Low back pain is not only a critical indication of medical rehabilitation, but also an important cause for loss from work (1). It has become a major public health problem worldwide and also an extremely common problem that occurs in above 80% of people (2). It is reported that the lifetime prevalence of low back pain is as high as 84%, and about 23% is chronic low back pain (3). Thus, the identification of risk and protective factors will be a key step to the prevention and treatment of low back pain.

The supplementation of omega-3 dietary was reported to reduce systemic inflammation and protect the progression of intervertebral disc (IVD) degeneration, a common cause of low back pain (4). Critically, a randomized controlled trial (RCT) demonstrated omega-3 dietary supplementation using fish oil significantly reduced low back pain (5). A recent RCT showed that eicosapentaenoic acid (EPA), one important omega-3 member, was effective for pain relief in adults with lower back pain (6). Although these results suggest an important role of omega-3 in the treatment of low back pain, the putative causal link between omega-3 and low back pain is still unclear.

Mendelian randomization (MR) is a method using specific genetic variants as a natural experiment to evaluate the putative causal relations between a potential exposure factor and outcome factor (7–9). Thus, we used a two-sample MR study to identify the putative causal link between plasma omega-3 levels and low back pain.

## Materials and Methods

### Ethics Approval and Consent to Participate

Our study was approved by the Ethics Committee of the Beijing Institute of Brain Disorders in Capital Medical University. This article contains human participants collected by several studies to report the large-scale genome-wide association study (GWAS) for omega-3 fatty acids from the CHARGE Consortium (10) and for low back pain https://gwas.mrcieu.ac.uk/datasets/finn-a-M13_LOWBACKPAIN. All participants gave informed consent in all the corresponding original studies, as described in the Methods.

### Study Design

**Figure 1:**
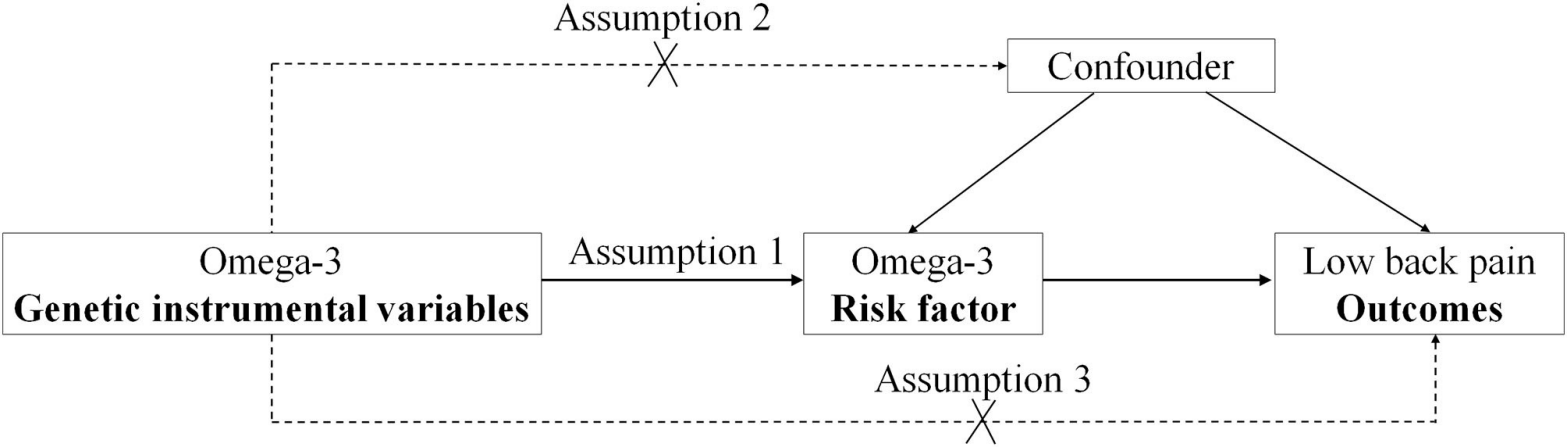
The design flow chart for the Mendelian randomization (MR) study. MR assumptions: assumption 1, 2, and 3. The solid line represents direct putative causal effects that plasma omega-3 genetic instrumental variants are reliably associated with I plasma omega-3 levels and influence the risk of low back pain through the plasma omega-3 in assumption 1. The dotted line represents that plasma omega-3 genetic instrumental variants are not associated with any measured and unmeasured confounders and do not influence the risk of low back pain through other pathways in assumptions 2 and 3, respectively.

In the two-sample MR study, three principal assumptions (11, 12) were described in Figure 1. Assumption 1 was that omega-3 fatty acid genetic instrument variants are reliably associated with the levels of plasma omega-3 fatty acids (exposure factor). Assumption 2 was that omega-3 fatty acid genetic instrument variants should undoubtedly be not associated with any confounders. Assumption 3 was that omega-3 fatty acid genetic instrument variants firmly influence the risk of outcome (low back pain) through exposure factor (the omega-3 fatty acids) but not through other pathways.

### Plasma Omega-3 Fatty Acid Genetic Instrumental Variants (IVs)

The plasma omega-3 fatty acid GWAS (*n* = 8,866 individuals from European ancestry) was reported by Lemaitre et al. (10). It is a well-known genome-wide association meta-analysis of omega-3 fatty acids from individuals of European ancestry (10). Six single nucleotide polymorphisms (SNPs) in Table 1 with *P* < 5 × 10^−8^ and no linkage disequilibrium (LD) (*R*^2^ < 0.001) were selected as the genetic IVs and broadly utilized in the MR analysis (13–15). Of six omega-3 SNPs, 3, 2, and 1 SNPs are for docosapentaenoic acid (DPA), eicosapentaenoic acid (EPA), and docosahexaenoic acid (DHA), respectively.

**Table 1:** Six plasma omega-3 genetic instrumental variants.

**SNP**	**EA**	**NEA**	**EAF**	**Beta**	**SE**	***p* val**	**Gene**
rs3798713	C	G	0.43	0.035	0.0051	1.93E-12	*ELOVL2*
rs174538	G	A	0.72	0.0834	0.0056	5.37E-58	*C11orf10*
rs780094	T	C	0.41	0.0167	0.0031	9.04E-09	*GCKR*
rs3734398	C	T	0.57	0.0404	0.0026	9.61E-44	*ELOVL2*
rs174547	T	C	0.33	0.0746	0.0026	3.79E-154	*FADS1*
rs2236212	G	C	0.57	0.1132	0.0143	1.26E-15	*ELOVL2*

*SNP, single-nucleotide polymorphism; EA, effect allele; NEA, non-effect allele; EAF, effect allele frequency; Beta, the regression coefficient based on omega-3 raising effect allele; SE, standard error; Six SNPs with p < 5 × 10^−8^ were selected as independent genetic instrumental variants*.

### Low Back Pain GWAS Dataset

The summary dataset of low back pain GWAS was found by searching GWAS ID: finn-a-M13_LOWBACKPAIN on the web https://gwas.mrcieu.ac.uk/datasets/. This GWAS consisting of 4,863 low back pain cases and 74,589 controls was identified in 2020. All individuals are from European ancestry. The demographic profiles about low back pain GWAS were summarized in Table 2.

**Table 2:** Genome-wide association study (GWAS) for low back pain.

**GWAS ID**	**Year**	**Trait**	**ncase**	**ncontrol**	**nsnp**	**Population**	**Sex**
finn-a-M13_LOWBACKPAIN	2020	Low back pain	4,863	74,589	16,152,119	European	Men and Women

*GWAS ID, Genome-wide association study identity; ncase, the number of low back pain case; ncontrol, the number of the control; nsnp, the number of single-nucleotide polymorphism*.

### Extracting the Omega-3 Fatty Acid Genetic IVs From low Back Pain GWAS Dataset

We successfully extracted the summary statistics corresponding to the six omega-3 fatty acid genetic IVs from low back pain GWAS. The summary about the association of the omega-3 genetic IVs in the low back pain GWAS dataset is shown in Table 3.

**Table 3:** Association of plasma omega-3 genetic instrumental variables (IVs) with low back pain GWAS.

**SNP**	**Exposure (plasma omega-3) GWAS**			**Outcome (low back pain) GWAS**c		
	**Beta**	**SE**	***p* val**	**Beta**	**SE**	***p* val**
rs174538	0.0834	0.0056	5.37E-58	−0.070	0.023	0.002
rs174547	0.0746	0.0026	3.79E-154	−0.059	0.022	0.007
rs2236212	0.1132	0.0143	1.26E-15	−0.016	0.022	0.472
rs3734398	0.0404	0.0026	9.61E-44	0.016	0.022	0.469
rs3798713	0.035	0.0051	1.93E-12	0.016	0.022	0.471
rs780094	0.0167	0.0031	9.04E-09	−0.001	0.022	0.969

*SNP, single-nucleotide polymorphism; EA, effect allele; NEA, non-effect allele; EAF, effect allele frequency; Beta, the regression coefficient based on plasma omega-3 raising effect allele; SE, standard error*.

### Pleiotropy Test

MR-egger_intercept and MR-PRESSO tests have been used to examine the pleiotropy and have previously been described (16). The TwoSampleMR R package was used to perform MR-egger_intercept and MR-PRESSO tests by the function “mr_pleiotropy_test” and “MR-PRESSO,” respectively. MR-egger_intercept and MR-PRESSO tests demonstrated that *p* values were.569 and.173, respectively. *P* ≥ 0.05 represents no significant pleiotropy of the omega-3 genetic IVs in low back pain GWAS. The summary of the pleiotropy test is shown in Table 4.

**Table 4:** Pleiotropy test of plasma omega-3 genetic IVs in GWAS for low back pain.

**Pleiotropy test**				**Heterogeneity test**					
**MR_Egger**			**PRESSO**	**MR Egger**			**IVW**		
**Intercept**	**SE**	***p* val**	***p* val**	**Q**	**Q_df**	**Q_pval**	**Q**	**Q_df**	**Q_pval**
0.018	0.029	0.569	0.173	9.405	4	0.052	10.309	5	0.067

*SE, standard error. p val ≥ 0.05 represents no significant pleiotropy. Q_pval ≥ 0.05 represents no significant heterogeneity*.

### Heterogeneity Test

MR_egger and inverse variance weighted (IVW) in Cochran's Q statistic have been broadly used to examine the heterogeneity and previously been described (17, 18). The TwoSampleMR R package was used to perform Cochran's Q statistic by the function “mr_heterogeneity.” MR_egger and IVW tests in Cochran's Q statistic demonstrated that *p* values were 0.052 and 0.067, respectively. *P* ≥ 0.05 represents no significant heterogeneity of the omega-3 genetic IVs in low back pain GWAS. The summary results of the heterogeneity test are shown in Table 4.

### MR Analysis

The TwoSampleMR R package was used to perform the MR analysis. The MR analysis was performed by the function “mr.” We selected four MR analysis methods, namely, MR-egger, weighted median, IVW, and Weighted mode (16, 19, 20). The effect size (beta) and (SE) correspond to 1 SD in the omega-3 levels. *P* < 0.05 represents the putative causal link between the omega-3 levels and low back pain. The summary results of MR analysis were shown in Table 5.

**Table 5:** The putative causal association of plasma omega-3 levels with low back pain.

**Method**	**nsnp**	**Beta**	**SE**	***p* val**	**OR**	**OR_lci95**	**OR_uci95**
MR Egger	6	−0.593	0.417	0.228	0.553	0.244	1.251
Weighted median	6	−0.436	0.195	0.025	0.646	0.441	0.947
IVW	6	−0.366	0.186	0.049	0.694	0.482	0.999
Weighted mode	6	−0.251	0.208	0.281	0.778	0.518	1.169

*IVW, Inverse variance weighted; nsnp, the number of single-nucleotide polymorphisms; Beta, the regression coefficient based on plasma omega-3 raising effect allele; SE, standard error; p < 0.05 represents the putative causal association of the increased levels of plasma omega-3 with low back pain; OR, Odds ratio; OR_lci95, Lower limit of 95% confidence interval for OR; OR_uci95, Upper limit of 95% confidence interval for OR*.

### Single SNP Effect Analysis

**Figure 2:**
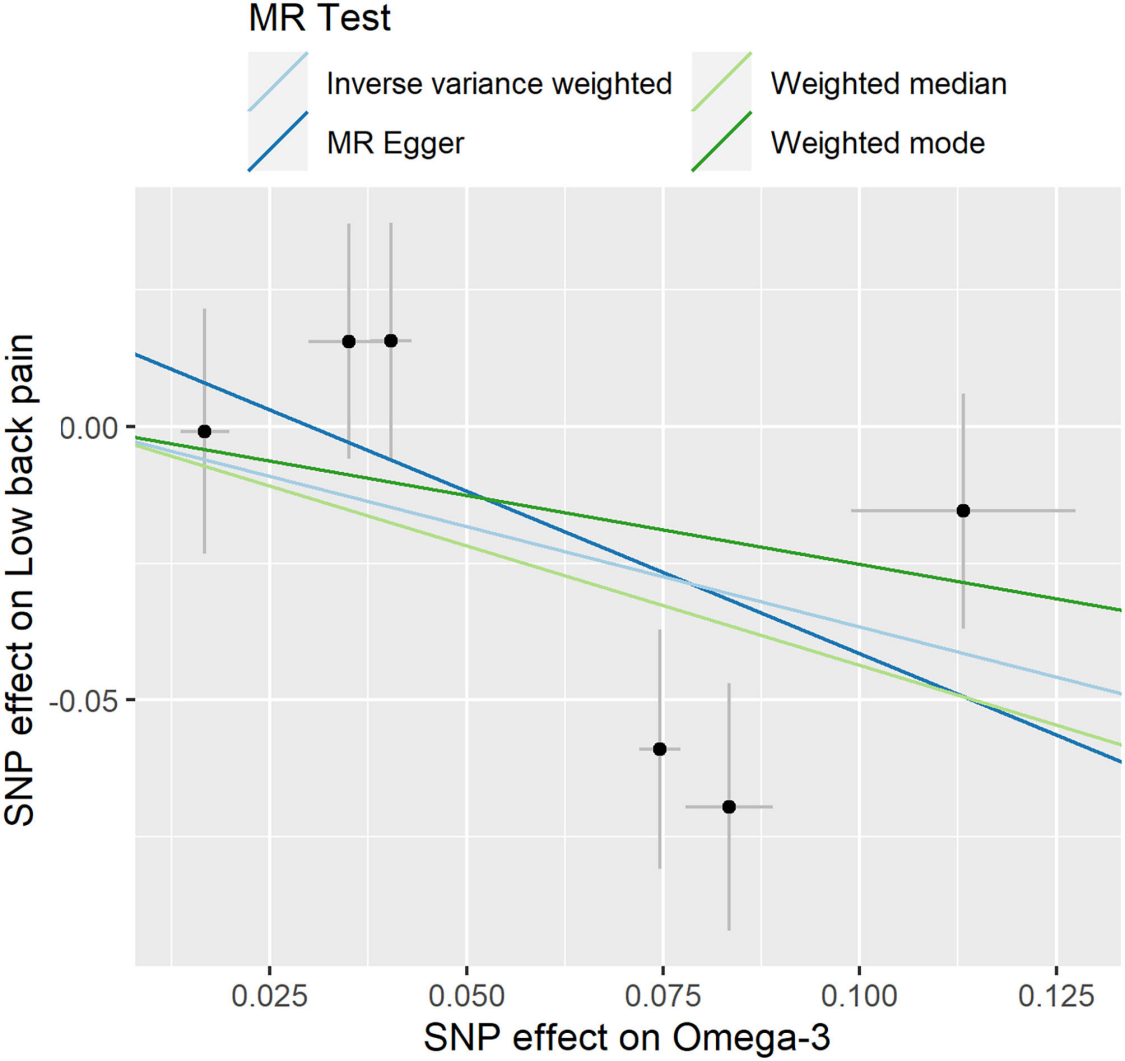
Individual estimates about the putative causal effect of omega-3 on low back pain. The x-axis shows the single nucleotide polymorphism (SNP) effect and SE on each omega-3. The y-axis shows the SNP effect and SE on low back pain. The regression line for MR Egger, Weighted median, inverse variance weighted (IVW), Simple mode, and Weighted mode is shown.

**Figure 3:**
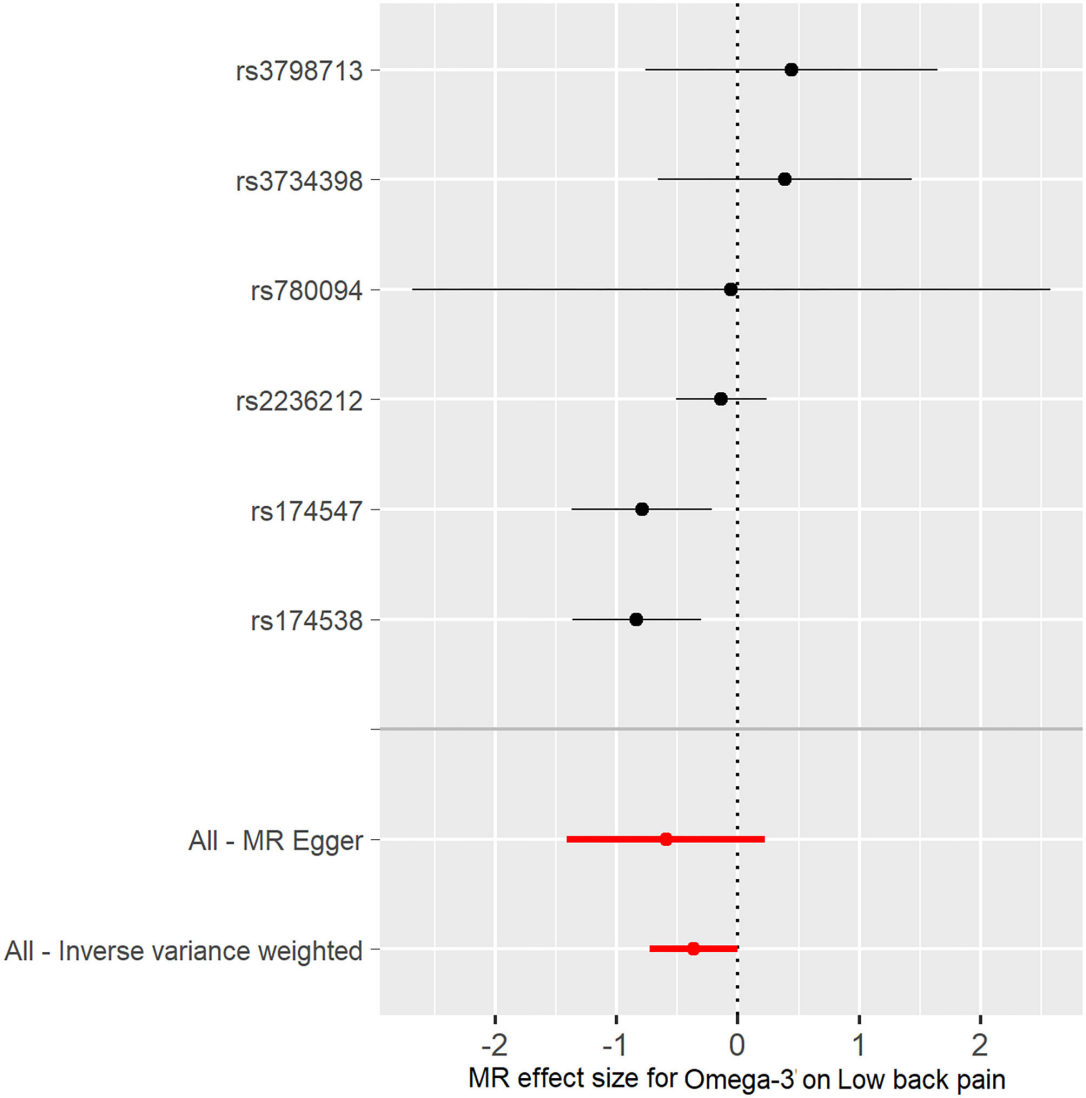
Forest plot of omega-3 associated with risk of low back pain. The x-axis shows the MR effect size for omega-3 on low back pain. The y-axis shows the analysis for each of the SNPs.

**Figure 4:**
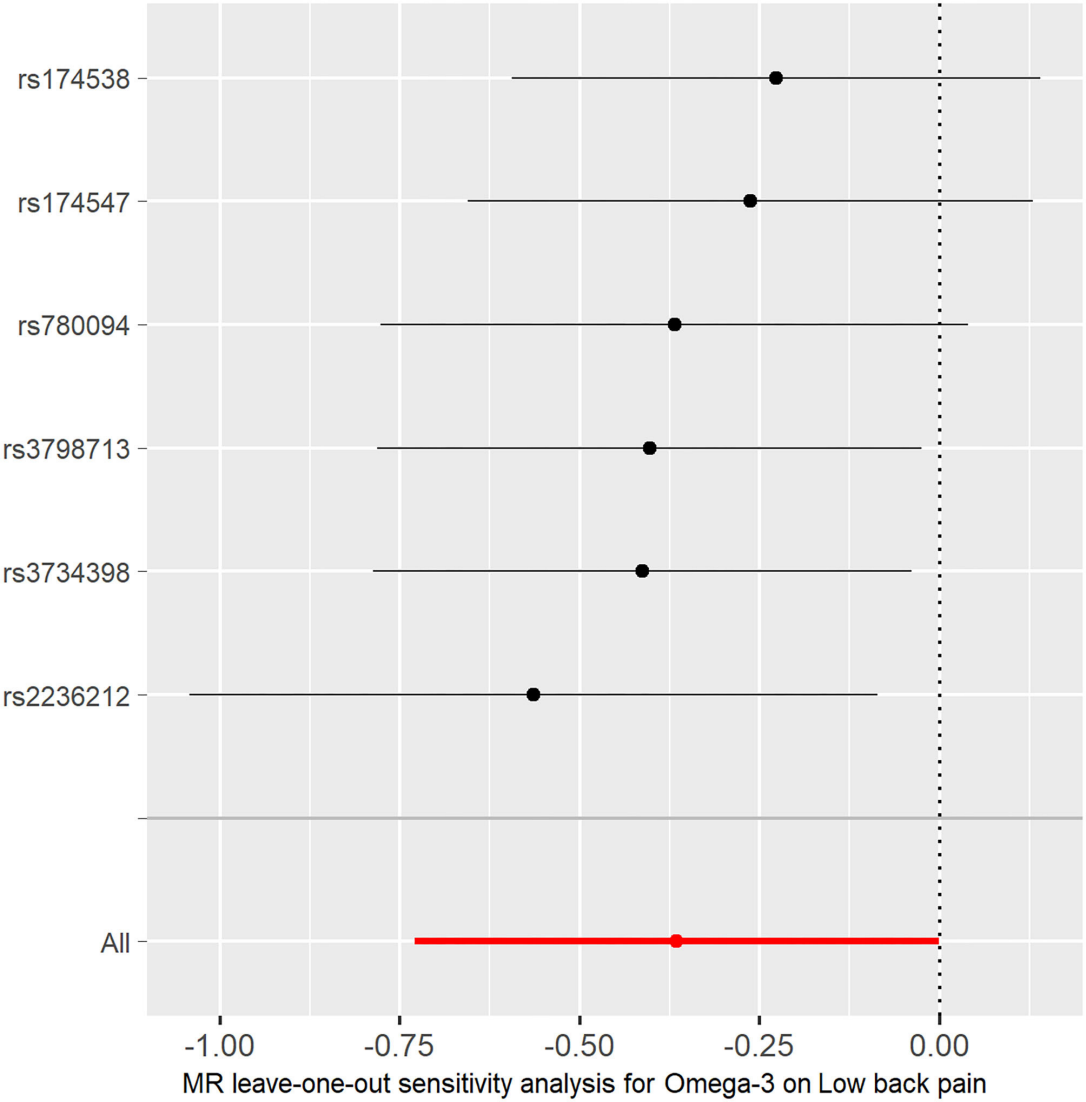
MR leave-one-out sensitivity analysis for the effect of omega-3 SNPs on low back pain. The x-axis shows MR leave-one-out sensitivity analysis for omega-3 on low back pain. The y-axis shows the analysis for the effect of leave-one-out of SNPs on low back pain.

In the TwoSampleMR R package, two functions, “mr” and “mr_scatter_plot,” were used to test the individual putative causal effect of plasma omega-3 levels on low back pain (Figure 2). To determine the single SNP effect size for omega-3 on low back pain, two functions, “mr_singlesnp” and “mr_forest_plot,” were used in the TwoSampleMR R package (Figure 3). To determine the single SNP bias of 11 independent plasma omega-3 genetic IVs in low back pain, two functions “mr_singlesnp” and “mr_leaveoneout_plot” in the TwoSampleMR R package were used to analyze the effect of leave-one-out (Figure 4).

## Results

### Pleiotropy and Heterogeneity Analysis

Six omega-3 fatty acid genetic IVs (Table 1) were successfully extracted from the low back pain GWAS dataset (Table 2). The association of six omega-3 fatty acid genetic IVs in the low back pain GWAS is shown (Table 3). Both MR-egger intercept and the MR-PRESSO test showed *p* > 0.05 (Table 4) suggesting no significant pleiotropy of Six omega-3 fatty acid genetic IVs in the low back pain GWAS. In addition, both MR egger and IVW in Cochran's *Q*-test showed *p* > 0.05 (Table 4) suggesting no significant heterogeneity of six omega-3 fatty acid genetic IVs in low back pain GWAS. Thus, all these selected omega-3 genetic IVs should be taken as the effective IVs in the MR analysis.

### MR Analysis

Expectedly, we found that as plasma omega-3 levels genetically increased, the risk of low back pain had a decreased trend using MR-egger (Beta = −0.593, *p* = 0.228; OR = 0.553) and weighted mode (Beta = −0.251, *p* = 0.281; OR = 0.778) (Table 5). This reduced trend was further proven by weighted median (Beta = −0.436, *p* = 0.025; OR = 0.646) and IVW (Beta = −0.366, *p* = 0.049; OR = 0.694) (Table 5). Our analysis suggested a putative causal association between genetically increased plasma omega-3 levels and the reduced risk of low back pain from European ancestries.

### Single SNP Effect Analysis

The individual MR estimates demonstrate that as the effect of a single SNP on omega-3 increased, the suppressive effect of a single SNP on low back pain increased, as determined using four MR analysis methods, namely, MR-egger, weighted median, IVW, and weighted mode (Figure 2). Each effect size analysis suggests that each effect of omega-3 SNPs on low back pain was robust (Figure 3). The MR leave-one-out sensitivity analysis suggested that removing a specific SNP of the six omega-3 SNPs did not change the results (Figure 4). Altogether, these results indicate that our data were robust without obvious bias.

## Discussion

Previous observational studies have suggested an important role of omega-3 in low back pain (4–6). In the present two-sample MR study, we established a putative causal link between plasma omega-3 levels and low back pain in the European population.

Pharmacologic treatments are fundamental for both acute and chronic low back pain (21). Acetaminophen and non-steroidal anti-inflammatory drugs (NSAIDs) have been shown effective for short-term relief (22–24). Omega-3 fatty acids are known to reduce inflammatory processes with a relatively benign side effect profile (4). Thus, omega-3 dietary supplementation has potential protective effects on the progression of spinal disc degeneration by reducing systemic inflammation (4).

Mendelian randomization (MR) study uses genetic variants to determine whether an observational association between a risk factor (e.g., omega-3) and an outcome (e.g., low back pain) is consistent with a putative causal effect (25). This approach retains the benefits of using genetic instruments for putative causal inference, such as avoiding bias due to confounding, while allowing for estimation of the different effects required for mediation analysis (26). Thus, a putative causal link between plasma omega-3 levels and low back pain in the European population is strong because of the advantages of the MR study.

Our MR study has several strengths. First, GWAS datasets for low back pain and omega-3 genetic IVs are from European ancestries. Thus, this removes the influence of population stratification. Second, omega-3 genetic IVs were chosen from several previous papers (13–15). Third, we used four different methods to prove independent omega-3 genetic SNPs as the effective IVs. Fourth, we used four MR analysis methods including MR-egger, weighted median, IVW, and weighted mode. Finally, we used three methods to demonstrate a single SNP effect and showed that omega-3 genetic IVs were robust without obvious bias.

Of course, our MR study also had many limitations. First, we only analyzed one GWAS for low back pain. We need much more GWAS datasets for low back pain to prove our conclusion. Second, we identified a putative causal link between omega-3 and low back pain in European ancestries. We need to expand our conclusion to other populations. Finally, the mechanisms by which genetically increased plasma omega-3 levels reduce the risk of low back pain in the European population need further be explored.

Omega-3 is present in several dietary supplement formulations including fish oil. A 1,000 mg fish oil supplement provides 180 mg EPA and 120 mg DHA. A previous randomized controlled trial (RCT) demonstrated that after 3 months of supplementation with 15 mL fish oil daily (550 mg EPA; 205 mg DHA), 36 girls aged 18–22 years had a marked reduction in low back pain (5). Based on this RCT, we recommended 3 g fish oil daily for 3 months to treat low back pain. Of course, more research on the recommended dosage of fish oil is needed using randomized controlled trials (RCTs).

In summary, our MR study suggested a putative causal association between genetically increased plasma omega-3 levels and reduced risk of low back pain in the European population. Thus, it is important for European patients with low back pain to take in omega-3 fatty acids.

## Data Availability Statement

The original contributions presented in the study are included in the article/supplementary material, further inquiries can be directed to the corresponding author/s.

## Ethics Statement

Our study was approved by the Ethics Committee of Beijing Institute of Brain Disorders in Capital Medical University. This article contains human participants collected by several studies to report the large-scale GWAS for plasma omega-3 fatty acids and for low back pain. All participants gave informed consent in all the corresponding original studies, as described in the Methods.

## Author Contributions

RW conceived and initiated the project. RW, SZ, and GZ analyzed the data and wrote the manuscript. All authors contributed to the interpretation of the results, critical revision of the manuscript, and approved the final version of the manuscript.

## Funding

This study was supported by grants from the National Natural Science Foundation of China (82071758, 31770956, and 81704151) and the Beijing Municipal Commission of Education (KM201710025014).

## Conflict of Interest

The authors declare that the research was conducted in the absence of any commercial or financial relationships that could be construed as a potential conflict of interest.

## Publisher's Note

All claims expressed in this article are solely those of the authors and do not necessarily represent those of their affiliated organizations, or those of the publisher, the editors and the reviewers. Any product that may be evaluated in this article, or claim that may be made by its manufacturer, is not guaranteed or endorsed by the publisher.

## Abbreviations

GWAS: Genome-wide association study
MR: Mendelian randomization
SNP: Single nucleotide polymorphism
IVW: Inverse variance weighted.
